# Near Surface Mounted Composites for Flexural Strengthening of Reinforced Concrete Beams

**DOI:** 10.3390/polym8030067

**Published:** 2016-03-03

**Authors:** Md. Akter Hosen, Mohd Zamin Jumaat, Ubagaram Johnson Alengaram, A. B. M. Saiful Islam, Huzaifa bin Hashim

**Affiliations:** 1 Department of Civil Engineering, Faculty of Engineering, University of Malaya, 50603 Kuala Lumpur, Malaysia; zamin@um.edu.my (M.Z.J.); johnson@um.edu.my (U.J.A.); huzaifaonearth@yahoo.com (H.b.H.); 2 Department of Construction Engineering, College of Engineering, University of Dammam, 31451 Dammam, Saudi Arabia; abm.saiful@gmail.com

**Keywords:** NSM composites, flexural behavior, cover separation, analytical model, crack behavior

## Abstract

Existing structural components require strengthening after a certain period of time due to increases in service loads, errors in design, mechanical damage, and the need to extend the service period. Externally-bonded reinforcement (EBR) and near-surface mounted (NSM) reinforcement are two preferred strengthening approach. This paper presents a NSM technique incorporating NSM composites, namely steel and carbon fiber-reinforced polymer (CFRP) bars, as reinforcement. Experimental and analytical studies carried out to explore the performance of reinforced concrete (RC) members strengthened with the NSM composites. Analytical models were developed in predicting the maximum crack spacing and width, concrete cover separation failure loads, and deflection. A four-point bending test was applied on beams strengthened with different types and ratios of NSM reinforcement. The failure characteristics, yield, and ultimate capacities, deflection, strain, and cracking behavior of the beams were evaluated based on the experimental output. The test results indicate an increase in the cracking load of 69% and an increase in the ultimate load of 92% compared with the control beam. The predicted result from the analytical model shows good agreement with the experimental result, which ensures the competent implementation of the present NSM-steel and CFRP technique.

## 1. Introduction

Strengthening of existing reinforced concrete (RC) structures is a necessity due to the destructive environmental conditions, increased service loads, as well as errors in design and during construction. Externally-bonded reinforcement (EBR) and near-surface mounted (NSM) reinforcement are the leading strengthening techniques used for strengthening existing RC structures [1,2,3,4,5]. The EBR technique [6,7] is widely implemented on site and extensive research has been carried out in this area [8,9,10,11]. However, the main drawback of this method is that it often suffers from premature debonding due to high shear stresses at the plate ends [12,13,14]. Several researchers have introduced end anchorages to solve this type of failure [15,16]. Moreover, externally-bonded steel or fiber-reinforced polymer (FRP) plates are vulnerable to thermal, environmental, and mechanical damage. On the other hand, the NSM strengthening technique [17] has a number of advantages in comparison to the EBR technique. In NSM strengthening the surrounding concrete protects the NSM bars or strips from thermal, environmental, and mechanical damage. Improved durability, stress-sharing mechanisms, and fatigue performance are other advantages due to the NSM reinforcement being placed inside the structural member [18].

Several experimental investigations on flexural behavior of RC beams strengthened with NSM bars or strips using FRP materials have been introduced [19,20,21,22,23]. However, FRP materials are highly expensive and are limited in the market. In addition, FRP reinforcement shows reduce ductility. Whereas, steel bars are less expensive, easily available with good long-term durability and bond performance [24], as well as sufficient ductility. Masonry buildings and arch bridges have also been strengthened with such pre-cut grooves and stainless steel bars [25]. This strengthening strategy came to be termed the NSM technique. Recently, Almusallam *et al.* [26] investigated the experimental and numerical behaviour of RC beams strengthened in flexure with NSM bars. They used steel bars and GFRP bars as NSM reinforcement. Either one or two NSM bars were placed in the beams, which presents sheared span-to-depth ratios of 5.73 with a groove size for strengthening as 30 mm × 30 mm. The NSM bars were found to promote the flexural capacity of the RC beams. A review of existing literature shows that the NSM technique with steel bar reinforcement is rarely used. The NSM steel technique may offer an effective alternative due to a number of advantages of steel bars over FRP bars mentioned earlier. Almusallam *et al.* [26] used NSM steel reinforcement in RC beams. However, the beams were highly under-reinforced, and the shear span-depth ratio and groove size were very large, which may have predisposed the beams to fail in a flexural state even after strengthening.

In this paper, RC beams strengthened with the NSM composites technique and their structural performance was investigated by subjecting the beams to static loading. The studied parameters include the number of NSM grooves, type of NSM strengthening materials (CFRP and steel bars), and NSM reinforcement ratios. Steel is used as the strengthening material to ensure a cost-effective solution which is also structurally efficient (US $315–320 per ton price [27] of steel bars and, on the other hand, US $10–15 per meter price [28] of CFRP bars). An analytical model was developed and used to predict maximum crack spacing and width, deflection, and concrete cover separation failure loads. Load, deflection, and strain data were analyzed to understand cracking behavior and failure modes of the tested beams. The test results showed that the NSM composites technique significantly improved the flexural capacity.

## 2. Experimental Program

### 2.1. Test Matrix

The experimental program consisted of six RC beam specimens. The first beam specimen was the control beam with no strengthening, and the remaining beam specimens were strengthened with steel and CFRP bars. Table 1 presents the various strengthening configurations used on beams in the experimental program.

### 2.2. Specimen Configuration

**Figure 1:**
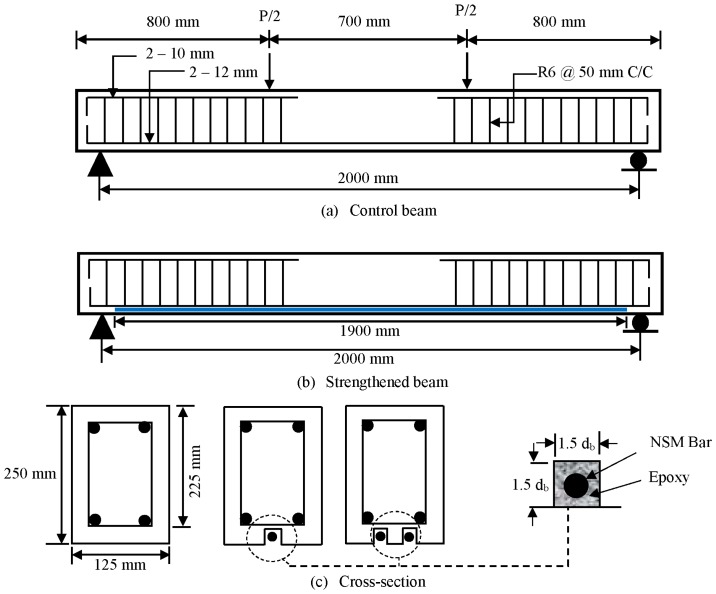
Beam specimen details.

The dimensions and reinforcement details of beam specimens are presented in Figure 1. The beams were designed as under-reinforced beams to initiate failure in a flexural state, in accordance with the ACI code [29]. The cross-sectional dimensions of the beams were 125 mm × 250 mm and the length of the beams was 2300 mm. The effective span and shear-span lengths of the beams were 2000 and 650 mm, respectively. Three types of steel bars, namely, 12, 10 and 6 mm in diameter, were used in constructing the beam specimens. The internal tension reinforcement of all beams consist of two deformed steel bars, 12 mm in diameter, which were bent ninety degrees at both ends to fulfil the anchorage criteria. In addition, two deformed steel bars of 10 mm diameter were used as hanger bars in the shear span zone. The shear reinforcement consisted of plain steel bars, 6 mm in diameter, distributed along the length of the beams as shown in Figure 1.

### 2.3. Material Properties

Beam specimens were cast using normal concrete. Crushed granite with a maximum size of 20 mm was used as coarse aggregate. Natural river sand was used as fine aggregate. Fresh tap water was used to hydrate the concrete mix during the casting and curing of the beams, cubes, prisms, and cylinders. The concrete mix design was in accordance with the DOE method [30] as shown in Table 2. The compressive strength of the concrete at 28 days was 40 MPa based on tests of three 100 mm × 100 mm × 100 mm concrete cubes. The compressive strength of concrete was calculated according to BS EN [31]. The yield strength of 6, 8, 10, 12 and 16 mm high-strength steel bars (E Steel Sdn. Bhd., Klang, Malaysia) are 520 MPa and the ultimate strengths were 570 MPa. The modulus of elasticity for all bars was 200 GPa. The ribbed CFRP bars (Haining Anjie Composite Material Co., Ltd., Haining, China) are used for strengthening in this study. The tensile strength and modulus of elasticity of 12 mm CFRP bar was 1850 MPa and 124 GPa respectively. Sikadur^®^ 30 (Sika Corporation, Lyndhurst, NJ, USA), an epoxy adhesive, was used to bond the strengthening materials to the concrete substrate. Sikadur^®^ 30 has two components, namely component A and component B. Component A is white in color, while component B is black. These two components were mixed in a ratio of 3:1 until a uniform grey color was achieved. The density was 1.65 kg/L at 23 °C after mixing. The bond strength according to DIN EN 24624 is 21 MPa with steel and 4 MPa with concrete. The compressive, tensile, shear strength, and the modulus of elasticity of the adhesive are shown in Table 3.

### 2.4. Strengthening Procedure

In the NSM-steel technique, strengthening bars were placed into grooves cut in the concrete cover of the RC beams and bonded using epoxy adhesive groove filler. The installation of the strengthening steel bars began with cutting groove with the dimensions 1.5 *d_b_* × 1.5 *d_b_* (where *d_b_* is the diameter of the tension reinforcement) into the concrete cover of the beam specimens at the tension face in the longitudinal direction. The groove was made using a special concrete saw with a diamond blade. A hammer and a hand chisel were used to remove any remaining concrete lugs and to roughen the lower surface of the groove. The groove was cleaned with a wire brush and a high-pressure air jet. The details of the groove are shown in Figure 1. The groove was half-filled with epoxy and then the steel bar was placed inside the groove and lightly pressed. This forced the epoxy to flow around the inserted steel bar. Epoxy was used to fill the groove and the surface leveled. The bonded length of the NSM steel bars was 1900 mm. In ensuring the epoxy achieved full strength, the beam was allowed to cure for one week.

### 2.5. Test Setup

**Figure 2:**
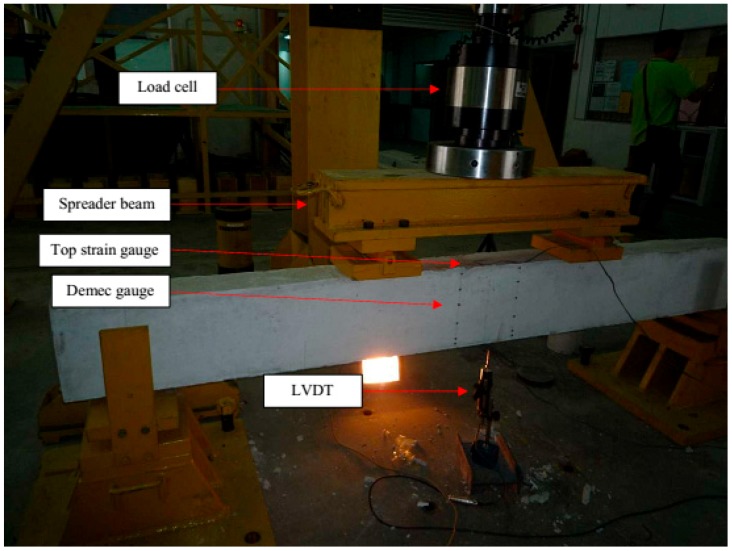
Experimental setup.

The instrumentation of the beams is presented in Figure 2. In measuring the deflection at beam midspan, one TML linear variable differential transducer (LVDT) was used. A number of gauges were used to measure strains. Two 5 mm strain gauges were attached to the middle of the internal tension bars. A 30 mm strain gauge was placed on the top surface of the beam at midspan. Demec gauges were attached along the depth of the beam at midspan. All beams were tested in four-point bending using an Instron Universal Testing Machine at heavy structural lab. The experiments were carried out using two controlling techniques. The first was load control, used for strain hardening. Commencing from the strain softening region, displacement control loading was maintained until failure. All data were recorded at 10 s intervals. The rate of the actuator was set to 5 kN/min during load control and 1.5 mm/min during displacement control. A Dino-Lite digital microscope was used to measure crack widths on the beams during testing.

## 3. Results and Discussion

The flexural capacities of tested beams were evaluated by a static four point bending test, causing the larger portion of the beams to take maximum stress. The key factors considered in this studies are cracking load, ultimate load, crack width and spacing, concrete compressive strain, tensile strain in the main steel reinforcement, sectional strain, and mode of failures. The experimental performance of all tested beams in terms of flexural load capacities are shown in Table 4.

### 3.1. Load-Deflection Behavior

**Figure 3:**
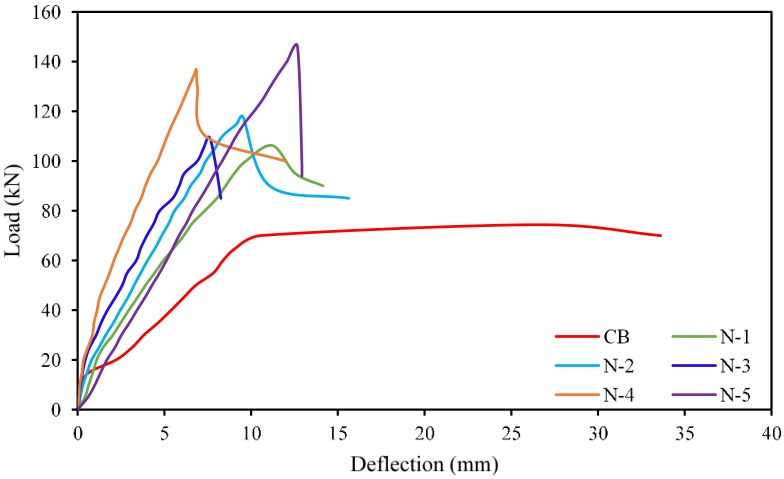
Load *vs.* deflection at midspan for all beams.

The load *versus* midspan deflection curves for beams CB, N-1, N-2, N-3, N-4, and N-5 are presented in Figure 3. All the strengthened beam specimens demonstrate a bi-linear response properties by cracking and ultimate stages. The cracking stage of the strengthened beam specimens follows a linear elastic shape identical to the control beam specimen. In this stage, the NSM bars have insignificant influence on the stiffness of the beam as observed in the load-deflection curves. However, a gradual reduction in deflection was observed for the strengthened beams. In addition, a significant increase in first cracking load was observed. The first cracking load increased by 27%, 33%, 52%, 69%, and 59% for N-1, N-2, N-3, and N-4, respectively, compared with the control beam (Table 4). In the second stage, cracking started at the maximum moment zone of the concrete cross-section. Due to increase in the applied load, the cracks become wider and fresh flexural cracks occured. After the yielding of the internal reinforcement, the NSM bars controls the crack widths until failure of the beams. All the strengthened beams experienced premature debonding failure. The ultimate load was reached during and just after the concrete cover separation, after which the imposed loading decreased rapidly. However, the control beam failed by yielding of the tension reinforcement, followed by concrete crushing *i.e.*, flexure failure.

### 3.2. Characteristics of Crack

**Figure 4:**
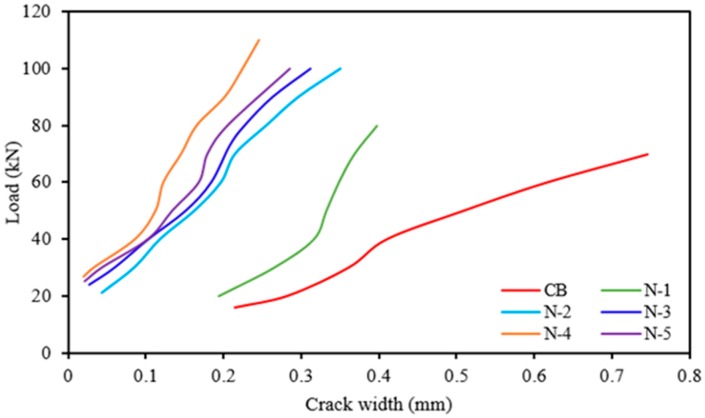
Load *vs.* crack width for all beams.

The load *versus* crack width of the beam specimens are presented in Figure 4. The first crack load of CB, N-1, N-2, N-3, N-4, and N-5 are 15.75, 20, 21, 24, 26.60 and 25 kN, respectively. All of the strengthened beams present higher first crack loads compared to the control beam. The use of NSM steel bars significantly improved the first crack load compared to the NSM CFRP bars. The total number of cracks on CB, N-1, N-2, N-3, N-4, and N-5 are 11, 15, 17, 19, 20, and 17, respectively. However, all strengthened beams show smaller crack spacing than the control beam. Thus, in this study, beams strengthened with NSM bars exhibit more and finer cracks with closer spacing than strengthened beams.

### 3.3. Modes of Failure

**Figure 5:**
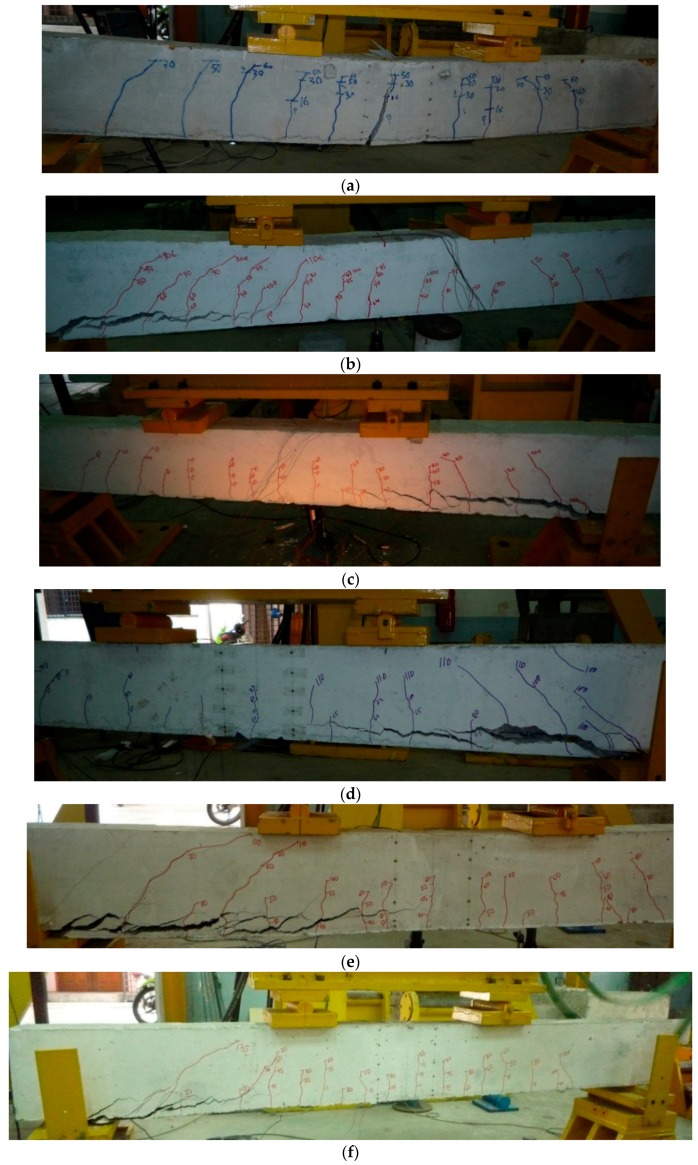
Modes of failure of all beams. (**a**) CB; (**b**) N-1; (**c**) N-2; (**d**) N-3; (**e**) N-4; and (**f**) N-5.

The failure modes of all the beams tested in this study are presented in Figure 5. At ultimate load, the control beam fails by concrete crushing, initially, and then by yielding of the tension steel reinforcement. As the external load increases, additional cracks are developed and flexural failure eventually takes place close to the midspan of the beam due to a spreading vertical crack. Nevertheless, all the strengthened beams (with NSM steel and CFRP bar) fail due to concrete cover separation. After the yielding of the internal main reinforcement, a shear crack initiates at the end of the NSM bars and the crack width rapidly increases. When shear stress and normal stress intersect, a concrete cover separation occurs, resulting in the premature debonding of the strengthened beams.

### 3.4. Concrete Compressive Strain in the Beam Surface

**Figure 6:**
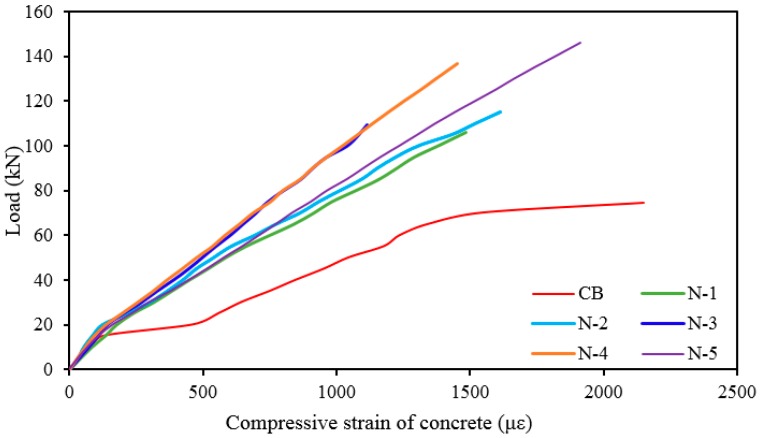
Load *vs.* compressive strain of concrete at midspan for all beams.

The load *versus* concrete compressive strain at the top fiber of the beams is presented in Figure 6. The concrete compressive strains of all strengthened beams are less than the concrete compressive strains of the control beam because of greater stiffness of the strengthened beams. All strengthened beams show linear variation in strain up to steel yielding. The increase in steel reinforcement in strengthened beams causes the magnitude of strains to decrease significantly. The greater reduction in strains for beam specimens with more strengthening shows the merit of the NSM-steel technique than NSM-CFRP. This can also be observed in the illustration.

### 3.5. Tensile Strain in Main Reinforcement

**Figure 7:**
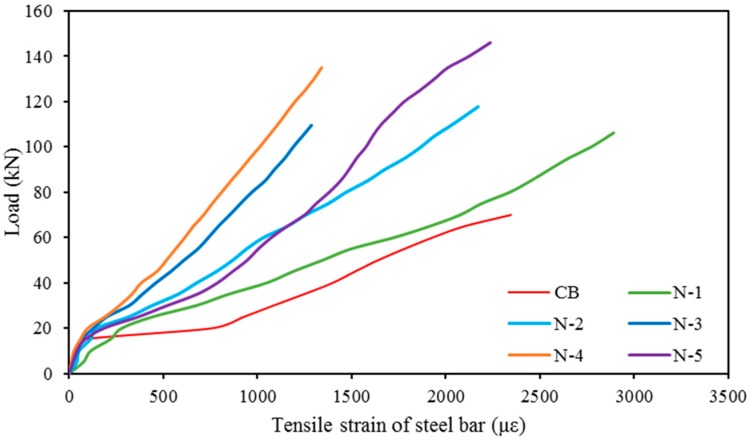
Load *vs.* tensile strain in main reinforcements at midspan for all beams.

Figure 7 shows the variation of imposing load with the measured strains in the internal main tension reinforcement during loading. The tension steel bar strains of all the strengthened beams are smaller than the tension steel bar strains of the control beam as the strengthened beams have elevated stiffness. After the first crack in a concrete section, the tensile stress occurs in the steel bars. Due to this, there is an abrupt increase in the strain in the tension steel bars after the first crack. This rate of increment was greater for the control beam than the strengthened beams, as the control beam offers larger crack widths.

### 3.6. Sectional Strain Profile

**Figure 8:**
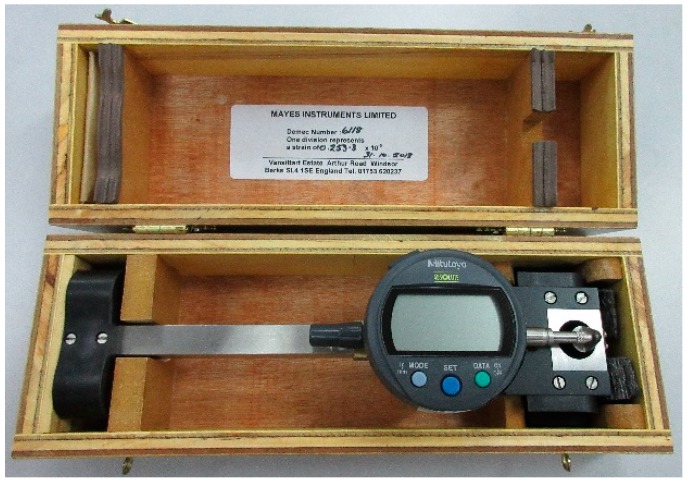
Digital extensometer for measure the sectional strain.

**Figure 9:**
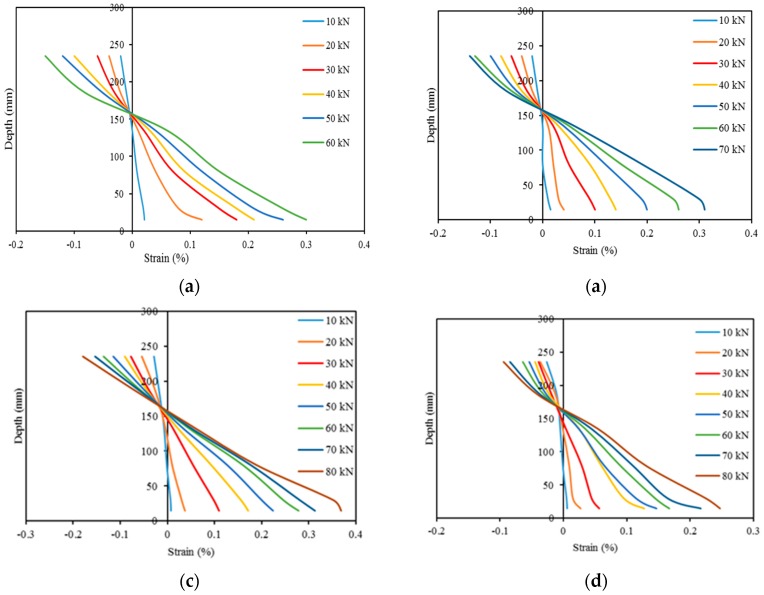

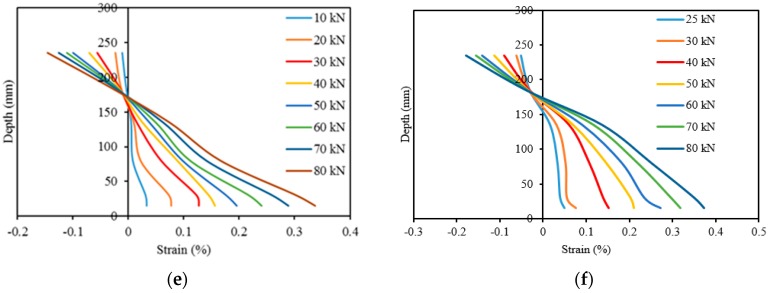
Sectional strain variation at mid-span during loading on strengthen beams. (**a**) CB; (**b**) N1; (**c**) N2; (**d**) N3; (**e**) N4; and (**f**) N5.

Demec gauges were attached along the depth of the beam at midspan (Figure 2) and measured the sectional strain by a digital extensometer (Figure 8). The variation of sectional strain in the concrete over the depth of the beams CB, N-1, N-2, N-3, N-4, and N-5 at different load levels are presented in Figure 9. The strain variation is linear at the commencement of loading (up to 30 kN) and the variation increases with higher load levels. For the strengthened beams, the strain deviation increases at the NSM bar level due to higher stress concentration. In the N3 specimen shows less transverse strain compared with other strengthened specimen owing to prompt concrete separation failure. The depth of the neutral axis and the pattern of strain variation in of the strengthened beam specimens are almost similar because of the similar failure mode of the beam specimens.

## 4. Parametric Study

### 4.1. The Effect of NSM Reinforcement Amount on Strengthening

**Figure 10:**
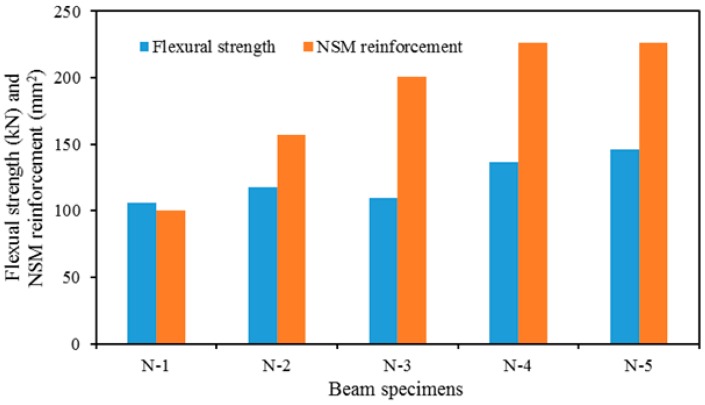
The effect of NSM reinforcement amount.

The influence of NSM reinforcement on the performance of the strengthened RC beams is presented in Figure 10. It was found that the increase number of NSM reinforcement offers higher load carrying capacity. The exception is beam N3, due to this specimen being strengthened with a single groove of NSM reinforcement. However, N-5 shows higher load carrying capacity due to NSM-CFRP bars.

### 4.2. The Effect of Type of NSM Reinforcement on Strengthening

**Figure 11:**
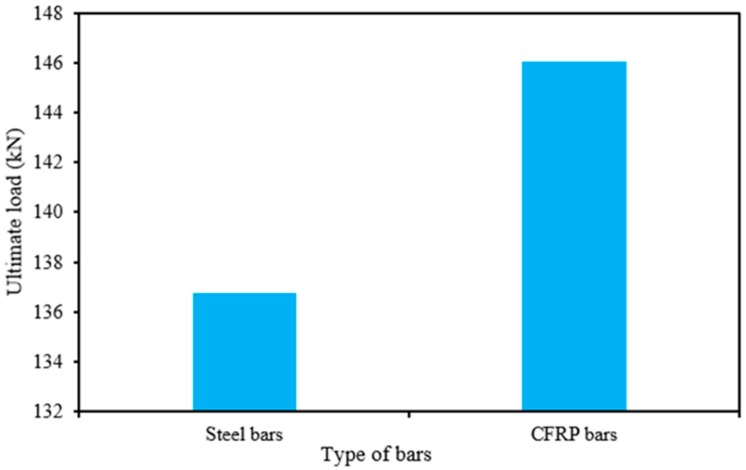
The effect of type of NSM reinforcement.

The effect of NSM reinforcement type on the performance by strengthening is presented in Figure 11. The CFRP bars increases the ultimate load carrying capacity more than 7% compared with the steel bars due to higher tensile strength. Therefore, the NSM-CFRP bars are used when needed to increase the ultimate load capacity of RC structural component.

### 4.3. The Effect of Number of NSM Groove

**Figure 12:**
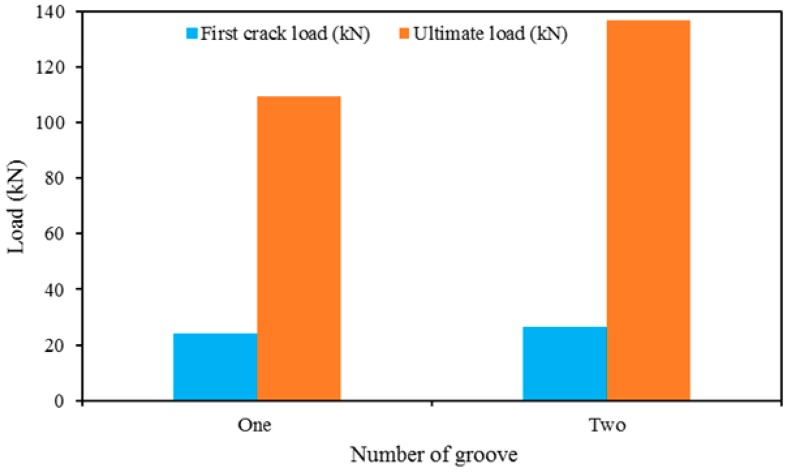
The effect of the number of grooves.

The influence of number of NSM groove on the NSM strengthening of RC beams are presented in Figure 12. The more or less similar amount of strengthening reinforcement (N-3 and N-4 for single and double grooves, respectively) is used to investigate this effect. The figure demonstrates that the number of grooves increases the first cracking and ultimate load, even though an increase in the number of grooves decreases the clear spacing and edge clearance of nearby grooves. The RC beam used in this study might not have enough width to provide two grooves. Therefore, the concrete separation failure may be accelerated due to stress overlap between adjacent grooves.

## 5. Analytical Study

Failure of a strengthened beam by concrete cover separation is governed by two factors [33]. The first factor is the initiation and propagation of the concrete cover separation. It contains two phases: (1) the formation of a shear crack at the end of the NSM bars, and (2) the spreading of the crack to the tension reinforcement level, which increases horizontally along the tension reinforcement level. The second factor is that the peak load of the strengthened beam is attained after concrete cover separation. This contains two phases; (1) the yielding of the main reinforcement in the shear flexure area, which decreases the shear resistance of the beam, and (2) the debonding of the concrete cover along the whole shear span zone. Prior to experimental works, analytical models are developed to predict the (a) concrete cover separation, (b) deflection, and (c) crack spacing. The following sections discuss the analytical models.

### 5.1. Concrete Cover Separation Model

**Figure 13:**
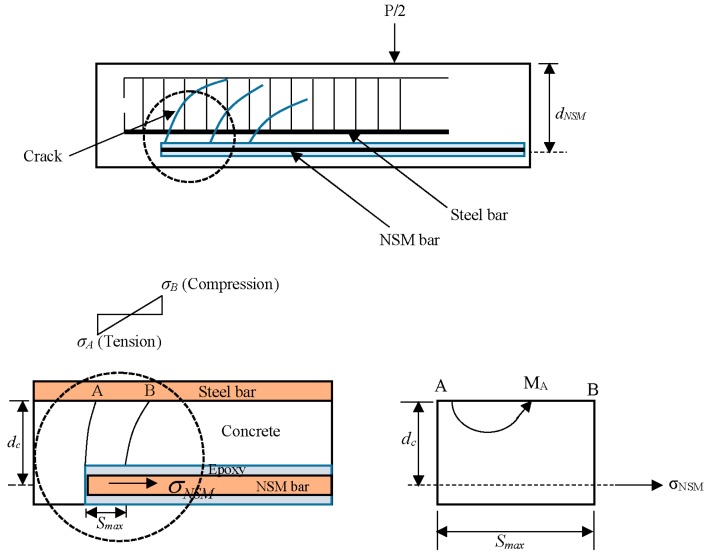
Distribution of stress in the NSM bars and concrete between the last two adjacent cracks at the end of the NSM bars.

The analytical model for predicting concrete cover separation is illustrated in Figure 13. The basic concept is that a concrete tooth is created between adjacent cracks [34] when external loading is imposed. In determining concrete cover separation failure loads, the prediction model depends on stabilized crack spacing.

The tensile stress at the critical point A ($\sigma_{A}$) can be calculated by bending moment:

(1) $$\sigma_{A}=\frac{M_{A}\left(S_{\max}/2\right)}{I_{A}}$$

where *I_A_* is moment of inertia of the section (*b* × *S*_max_):

(2) $$I_{A}=\frac{b\times {S_{\max}}^{3}}{12}$$

and

(3) $$M_{A}=\sigma_{NSM}A_{NSM}d_{c}$$

(4) $$\sigma_{A}=\frac{6A_{NSM}d_{c}}{b{S_{\max}}^{2}}\sigma_{NSM}$$

with

(5) $$\sigma_{NSM}=n_{NSM}\frac{d_{NSM}-y}{I_{cr}}M_{B}$$

(6) $$n_{NSM}=\frac{E_{NSM}}{E_{c}}$$

(7) $$E_{c}=4700\sqrt{{f_{c}}^{\prime }}$$

where, *M_A_* is the moment at point A, *b* is beam width, *S*_max_ is the maximum crack spacing, $A_{NSM}$ is the area of the side NSM reinforcement, dc is the distance between the centre of gravity of the NSM bars and the surface of the main steel bars, $\sigma_{NSM}$ is the stress in NSM bar, $n_{NSM}$ is the modular ratio of the NSM reinforcement, $E_{NSM}$ is Young’s Modulus of the NSM reinforcement, $E_{c}$ is Young’s Modulus of the concrete, *d_NSM_* is the distance between the center of gravity of the NSM bars and the concrete compression fiber, y is the depth of the neutral axis, *I_cr_* is the cracking moment of inertia, *f’_c_* is the compressive strength of concrete and *M_B_* is the moment at point B.

(8) $$y=\frac{-(nA_{s}+n_{NSM}A_{NSM})+\sqrt{{\left(nA_{s}+n_{NSM}A_{NSM}\right)}^{2}+2.b\left(nA_{s}d+n_{NSM}A_{NSM}d_{NSM}\right)}}{b}$$

(9) $$n=\frac{E_{s}}{E_{c}}$$

(10) $$I_{cr}=\frac{by^{3}}{3}+nA_{s}{\left(d-y\right)}^{2}+n_{NSM}A_{NSM}{\left(d_{NSM}-y\right)}^{2}$$

where, *n* is the modular ratio.

From Equations (4) and (5) the following equation was obtained:

(11) $$\sigma_{A}=\frac{6n_{NSM}A_{NSM}d_{c}}{b{S_{\max}}^{2}}\frac{\left(d_{NSM}-y\right)}{I_{cr}}M_{B}$$

Concrete cover separation occurs when the tensile strength at point A attains the ultimate tensile strength of the concrete *f_ct_* and the bending moment at point B (*M_B_*) is heading towards the beam failure load:

(12) $$M_{B}=\frac{f_{ct}I_{cr}b{S_{\max}}^{2}}{6n_{NSM}A_{NSM}d_{c}\left(d_{NSM}-y\right)}$$

### 5.2. Deflection Prediction Model

The deflection prediction of flexural RC member can be calculated using the effective moment of inertia (*I_e_*) [35], which is interpolation between the uncracked and fully cracked state.

(13) $$I_{e}=\frac{I_{cr}}{1-\eta {\left(\frac{M_{cr}}{M_{a}}\right)}^{2}}\leq I_{g}$$

(14) $$\eta =1-\frac{I_{cr}}{I_{g}}$$

(15) $$M_{cr}=\frac{f_{r}I_{g}}{y_{t}}$$

(16) $$I_{g}=\frac{bh^{3}}{12}$$

(17) $$f_{r}=0.70\sqrt{{f_{c}}^{\prime }}$$

where, $I_{g}$ is the gross moment of inertia, $M_{cr}$ is the cracking moment, $M_{a}$ is the service moment, $f_{r}$ is the modulus of rupture of concrete, $y_{t}$ is the distance between the extreme tension fiber of concrete and the neutral axis.

Therefore, the midspan deflection of RC beam specimens in this study was estimated [36] using the following equation:

(18) $$\Delta =\frac{\left(P/2\right)L_{a}}{24E_{c}I_{e}}\left(3L^{2}-4{L_{a}}^{2}\right)$$

where, Δ is the deflection at midspan, *P* is the predicted load, *L* is the total span length and *L*_a_ is the shear span length.

### 5.3. Crack Spacing Prediction Model

The maximum flexural crack spacing is reliant on the modular ratio and the position of the neutral axis of the composite section. Thus, to predict the crack spacing for the composite strengthened beams in this study the following analytical model [37] was used:

(19) $$S_{\max}=3.4c+0.425k_{1}k_{2}\frac{\varphi }{\rho_{eff}}$$

(20) $$\rho_{eff}=\frac{A_{s}+n_{NSM}A_{NSM}}{A_{ceff}}$$

(21) $$A_{ceff}=\min\left\{\begin{array}{l} 2.5\times b\times c\\ b\times (h-y)/3 \end{array}\right\}$$

where c is the concrete cover, *k*_1_ is the bond coefficient (0.80 and 1.6 for high bond and plain rebar, respectively), *k*_2_ is the strain distribution coefficient (0.50 and 1.0 for bending and pure tension, respectively), φ is the diameter of the rebar, $\rho_{eff}$ is the effective reinforcement ratio, *A*_s_ is the area of the tension steel, $A_{ceff}$ is the area of the concrete in tension, and h is the depth of the beam. Other symbols represent their usual meanings as given before.

### 5.4. Crack Width Prediction Model

The crack width approach [37] used to explain the cracking behavior of reinforced concrete is to consider the cracking of an RC beams, which is subjected to bending in a static condition. The crack width (*C*_w_) is expressed by the following equations:

(22) $$C_{w}=S_{\max}\left(\varepsilon_{sm}-\varepsilon_{cm}\right)$$

(23) $$\varepsilon_{sm}-\varepsilon_{cm}=\frac{\sigma_{s}}{E_{s}}-\frac{k_{t}f_{r}\left(1+n\rho_{eff}\right)}{E_{s}\rho_{eff}}\geq 0.6\frac{\sigma_{s}}{E_{s}}$$

(24) $$\sigma =\frac{\left(P/2\right)L_{a}}{A_{s}\left(d-\frac{y}{3}\right)+A_{NSM}\left(d_{NSM}-\frac{y}{3}\right)}$$

where $\varepsilon_{sm}$ is the tension reinforcement mean strain, $\varepsilon_{cm}$ is the mean strain between the concrete and cracks, σ is the tensile stress (cracked section), and $k_{t}$ = 0.6 for short-term loading or 0.4 for sustained loading. Other symbols represent their usual meanings as given before.

## 6. Verification of Experimental Results

The structural characteristics obtained from the experiments were compared with the predicted results as acquired from the above presented analytical models in order to clarify the authenticity of the investigation. It is worth mentioning that along with the experimental study, the analytical models are also useful tools for predicting the behavior of composite structural elements. To verify both approaches, the concrete cover separation failure load, deflection, and maximum crack spacing were evaluated.

### 6.1. Validation of Ultimate Loading

**Figure 14:**
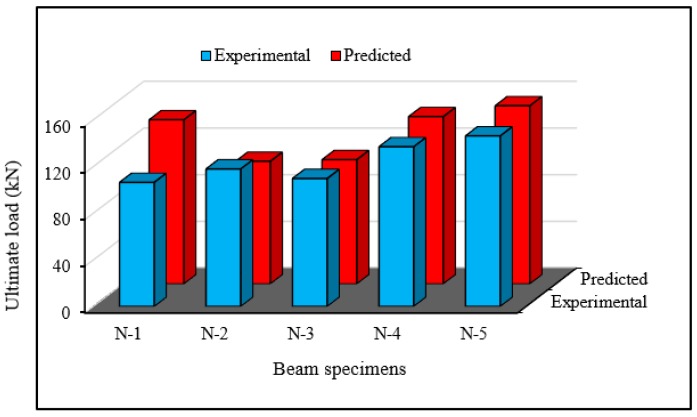
Comparison of experimental and predicted failure loads.

Figure 14 presents a comparison between the experimental and predicted failure loads of the strengthened beams. The results in the table show that the analytical predicted loads are mostly very similar to the achieved experimental loads. Except for beam N-1, the beams N-2, N-3, N-4 and N-5 reveal a very good agreement for the failure load. As the crack spacing for beam N-1 was higher in the predicted model, a major upturn in ultimate load was seen, which is desirable due to cracking behavior. However, the overall evaluation of ultimate load in both techniques is acceptable as the average deviation is trivial.

### 6.2. Validation of Ultimate Deflection

**Figure 15:**
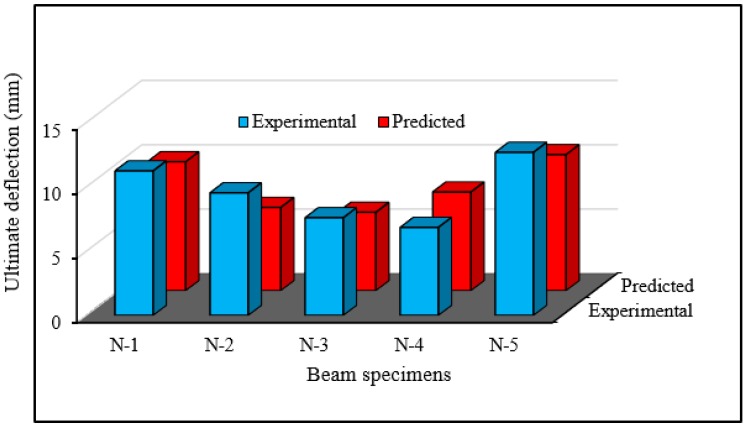
Comparison of experimental and predicted deflection.

The comparison between the ultimate deflection from the experimental tests and the predicted model is shown in Figure 15. The ratio of the average predicted ultimate deflection to the experimental deflection is 1.05. This phenomenon illustrates only a 5% variation in deflection. The results indicate that the proposed deflection prediction model is an effective tool for predicting the deflection of RC beams strengthened in flexure using steel bars.

### 6.3. Validation of Maximum Crack Spacing

**Figure 16:**
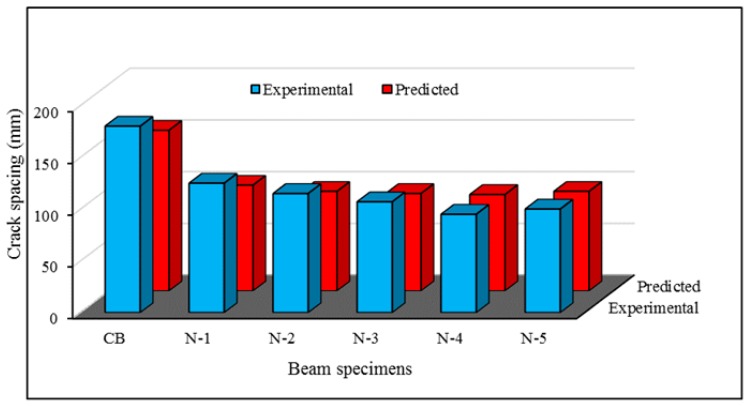
Comparison of experimental and predicted crack spacing.

Figure 16 presents the comparison between the maximum crack spacing from the experimental tests and the predicted model. The predicted and experimental results show reasonable agreement for crack spacing in the heavily-loaded composite beams.

### 6.4. Validation of Maximum Crack Width

**Figure 17:**
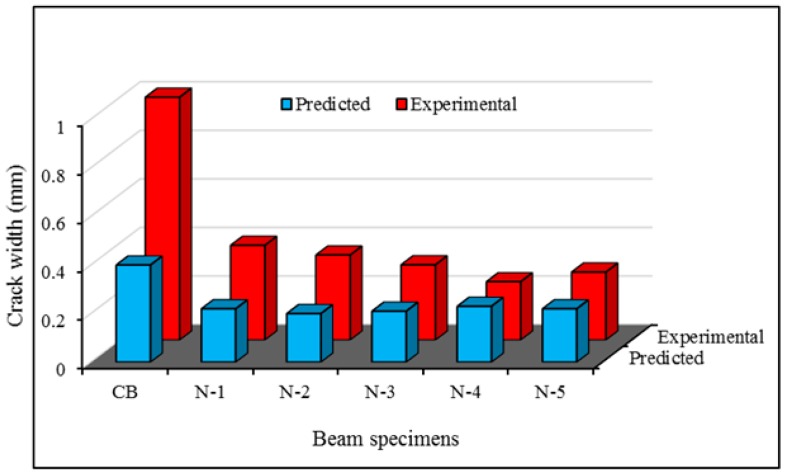
Comparison of experimental and predicted crack width.

Figure 17 shown the comparison between the crack width from the experimental tests and the predicted model. The predicted and experimental results demonstrate realistic agreement for crack width in all specimens, except the control specimen, due to higher crack spacing.

## 7. Conclusions

Based on the experimental and analytical study conducted, the effect of different variables on the flexural strength of RC beams strengthened with NSM steel and CFRP bars have been presented in this paper. The influence of varying NSM reinforcement ratios on the strengthened beams is studied. The flexural capacity, deflection, mode of failure, concrete top fiber strain, tension steel strain, and crack width of each of the tested beams were analyzed. The following conclusions can be drawn:

- The NSM strengthening technique with steel bars is a very cost effective method to improve the flexural strength of RC beams.
- The strengthened beam specimens expressed a bi-linear response to loading, characterized by cracking and ultimate stages. The cracking stage of the strengthened beams followed a linear elastic shape identical to the control beam.
- The NSM steel or CFRP bar technique increased the first crack load. Strengthened beams exhibited smaller crack spacing than the control beam. Thus, beams strengthened with NSM bars develop more and finer cracks with closer spacing than unstrengthened beams.
- Increasing the amount of NSM steel reinforcement from 100 to 226 mm^2^ improves the first crack load from 27% to 69% and the ultimate load capacity from 43% to 84%, respectively. NSM-CFRP increases the first cracking and ultimate load of 59% and 92%, respectively. After the yielding of internal reinforcement, NSM reinforcement controls crack widths until failure of the beam.
- The strengthened beams failed in premature debonding. The ultimate load was reached in the instant of debonding and directly afterwards, after which imposed loading decreased rapidly. The predicted results from the analytical models complied very well with the experimental results.

## Figures and Tables

**Table 1:** Test matrix.

Beam ID	Strengthening bars		NSM strengthening materials
	Number	Diameter (mm)	
CB	Control beam (Unstrengthened)		
N-1	2	8	Steel bars
N-2	2	10	
N-3	1	16	
N-4	2	12	
N-5	2	12	CFRP bars

**Table 2:** Concrete mix design.

Slump (mm)	W/C Ratio	Quantity (kg/m^3^)			
		Cement	Coarse aggregate	Fine aggregate	Water
45	0.50	420	892	888	224

**Table 3:** Properties of Sikadur^®^ 30 [32].

Properties	Strength (MPa)
Compressive strength	95
Tensile strength	31
Shear strength	19
Modulus of elasticity	11,200

**Table 4:** Flexural performance.

Specimens	First cracking load (kN)	Increase in first cracking load (%)	Ultimate load (kN)	Increase in ultimate load (%)
CB	15.75	–	74.37	–
N1	20.00	26.98	106.24	42.85
N2	21.00	33.33	117.75	58.33
N3	24.00	52.38	109.56	47.32
N4	26.60	68.89	136.75	83.88
N5	25.00	58.73	143.03	92.32
